# Study Protocol: Partners at Meals Telehealth Intervention for Caregivers of Persons With Dementia

**DOI:** 10.1093/geroni/igaa057.889

**Published:** 2020-12-16

**Authors:** Suparna Qanungo, Elaine Amella Krug, Kelly Martin, Martina Mueller, Mohan Madisetti, Teresa Kelechi

**Affiliations:** Medical University of South Carolina, Charleston, South Carolina, United States

## Abstract

Background: As the aging population continues to increase, it is estimated that persons with dementias (PWDs) will reach 13 million by 2050. Lack of caregiver skills related to mealtime planning and the ability to cope with dysfunctional behaviors are well-documented factors that influence nutritional outcomes for PWDs, leading to social isolation, and negatively impacting their home stay. The aim of this study protocol is to test the effectiveness of a train-the-trainer program in which non-paid volunteers in respite care centers deliver a telehealth mealtime intervention for caregivers of PWDs, Partners at Meals. The program is based on the C3P Model of Changing the Place, People and Person. Methods: A cluster-randomized controlled trial with parallel mixed methods evaluation processes is being conducted. Caregivers and PWD dyads, receiving respite services are randomized to receive Partners at Meals or enhanced-usual-care for six months. Within the intervention group, dyads are partnered with a C3P-trained volunteer who works with caregivers to devise monthly mealtime plans. Under enhanced-usual-care, dyads receive standardized educational materials modified from The Savvy Caregiver Program for Alzheimer’s caregiving. Primary outcomes include weight, calorie, protein and fluid intake of the PWDs and quality of life of the caregiver. Respite center administrators, program directors, volunteers and caregivers are evaluated for intervention fidelity, acceptability and sustainability. Implications: In this trial, we lay the groundwork to examine effectiveness and sustainability of a train-the-trainer telehealth program that could be widely disseminated for managing mealtimes in-the-home, while promoting quality of life of both the caregiver and PWDs.

